# Proteomics and ultrastructural analysis of *Hermetia illucens* (Diptera: Stratiomyidae) larval peritrophic matrix

**DOI:** 10.1186/s12953-021-00175-x

**Published:** 2021-04-09

**Authors:** Yu-Bo Lin, Jing-Jing Rong, Xun-Fan Wei, Zhuo-Xiao Sui, Jinhua Xiao, Da-Wei Huang

**Affiliations:** 1 Key Laboratory of Zoological Systematics and Evolution, Institute of Zoology, Chinese Academy of Sciences, Beijing, 100101 China; 2 University of Chinese Academy of Sciences, Beijing, 100049 China; 3 Institute of Entomology, College of Life Sciences, Nankai University, Tianjin, 300071 China

**Keywords:** *Hermetia illucens*, Liquid chromatography-tandem mass spectrometry, Midgut, Peritrophic matrix, Proteomic analysis, Scanning electron microscopy

## Abstract

**Background:**

The black soldier fly (*Hermetia illucens*) has significant economic potential. The larvae can be used in financially viable waste management systems, as they are voracious feeders able to efficiently convert low-quality waste into valuable biomass. However, most studies on *H. illucens* in recent decades have focused on optimizing their breeding and bioconversion conditions, while information on their biology is limited.

**Methods:**

About 200 fifth instar well-fed larvae were sacrificed in this work. The liquid chromatography-tandem mass spectrometry and scanning electron microscopy were employed in this study to perform a proteomic and ultrastructural analysis of the peritrophic matrix (PM) of *H. illucens* larvae.

**Results:**

A total of 565 proteins were identified in the PM samples of *H. illucen*, of which 177 proteins were predicted to contain signal peptides, bioinformatics analysis and manual curation determined 88 proteins may be associated with the PM, with functions in digestion, immunity, PM modulation, and others. The ultrastructure of the *H. illucens* larval PM observed by scanning electron microscopy shows a unique diamond-shaped chitin grid texture.

**Conclusions:**

It is the first and most comprehensive proteomics research about the PM of *H. illucens* larvae to date. All the proteins identified in this work has been discussed in details, except several unnamed or uncharacterized proteins, which should not be ignored and need further study. A comparison of the ultrastructure between *H. illucens* larval PM and those of other insects as observed by SEM indicates that the PM displays diverse textures on an ultra-micro scale and we suscept a unique diamond-shaped chitin grid texture may help *H. illucens* larval to hold more food. This work deepens our understanding of the molecular architecture and ultrastructure of the *H. illucens* larval PM.

**Supplementary Information:**

The online version contains supplementary material available at 10.1186/s12953-021-00175-x.

## Background

The peritrophic matrix (PM) is an acellular, semi-permeable structure lining the digestive tracts of certain invertebrates to encase food particles, as first reported in lepidopteran larvae [1, 2]. The original name was “peritrophic membrane” because of its appearance, but this term has been gradually replaced by “peritrophic matrix” in past decades; not only to avoid the misleading term “membrane” reserved in biology for lipid bilayers, but also to emphasize that the PM is actually an extracellular matrix with complex properties [3]. There are two types of PM: Type I PM is a multi-layered sleeve secreted by the epithelial cells along the entire midgut, and primarily observed in larval Lepidoptera and adult, blood-feeding Diptera. Secretion of Type I PM is mostly food-stimulated, as in female mosquitoes, but it can also be formed continuously, as in locusts. The more common, Type II PM is a highly ordered, 1–3 layers, sleeve-like structure secreted by a group of cells at the junction of the foregut and midgut. The physiological significance of these different types of PM is unclear [4, 5]. The PM is thought to be homologous to mucus secretions in the mammalian digestive tract [6], acting as a physical barrier to protect the midgut from rough food particles and digestive enzymes [7]. In addition, the PM can compartmentalize the midgut, promote digestion [8], neutralize toxic compounds [9], and act as an anti-infective barrier [10]. To understand how the PM performs these essential functions, one must study its molecular architecture [11].

The main components of the PM are protein and chitin. Chitin is a polysaccharide polymer of N-acetylglucosamine whose chains form strong, crystal microfibers by hydrogen bonding [12–14]. The PMs of different insects exhibit structural differences adapted to different food sources or physiological and immune challenges. In the typical microfiber arrangement, about 10 chitin microfibers are arranged in parallel to form a microfiber bundle. These bundles intersect to form a 60° or 90° grid structure, or they align in random directions to form a dense network structure. The pores of these grid structures are filled with many different proteins and carbohydrates.

PM-associated proteins can be divided into four classes according to their extractability [2]. The class I PM-associated proteins can be easily eluted from the PM with physiological buffers, such as many digestive enzymes. The class II PM-associated proteins can be removed with a mild detergent such as SDS to disrupt the weak ionic interactions. The class III proteins are tightly bound to the PM and require a strong denaturing agent such as urea to be extracted. The class IV proteins are thought to be covalently bound to the PM and so cannot be extracted by the above three methods. Proteins in the PM can also be divided into structural proteins, or peritrophins, and non-structural proteins. Structural proteins associate with the chitin scaffold and modify the integrity, elasticity, and permeability of the PM [15]. They usually contain more than one chitin-binding domain (CBD). Non-structural proteins are hydrolases and chitin-modifying enzymes, which regulate the structure of the PM, such as changing the pore size and permeability of the PM to adapt to physiological activities [16, 17]. In addition, many proteomic studies have shown that a large number of digestive enzymes are embedded in the PM, suggesting that the PM serves as a scaffold to which digestive enzymes associate [18, 19].

This paper examines the PM of the black solder fly (BSF; *Hermetia illucens* Linnaeus; Diptera: Stratiomyidae). BSF is thought to originate in the America and is currently widely spread in tropical, subtropical, and temperate regions [20]. Adults do not need to feed, do not bite, and are not disease vectors. The larvae have great potential utility in industry. 1) BSF larvae are a suitable protein source for poultry, swine, and several valuable fish species [21–23]. 2) BSF larvae are a potential source of bioactive substances like antimicrobial peptides, and enzymes such as cellulose-, chitin-, and lignin-degrading enzymes [24]. 3) BSF larvae are ideal for bioconversion, capable of efficiently transforming low-quality biomass like organic waste, kitchen waste, and agricultural by-products into high-quality protein [20, 25]. How they are able to digest so much so well is unknown. Most studies on BSF in recent decades have focused on the optimization of breeding conditions and the rearing of the larvae, but information on their biology is very limited [26–29].

In this work, mass spectrometry and scanning electron microscopy were employed to analyze the proteome and ultrastructure of the PM, to produce a more comprehensive understanding of the molecular architecture and ultrastructure of this essential structure.

## Materials and methods

### BSF larvae rearing and isolation of larval PM

BSF larvae were kept in an artificial climate chamber at a temperature of 27 °C, relative humidity of 70–80%, and a photoperiod of 12 h: 12 h light:dark. All the larvae were fed with commercially available wheat bran and water. About 200 fifth instar larvae were divided into three groups and sacrificed in this work. The larvae were anesthetized on ice, the larval epidermis was cut open, and the midgut was taken out. The PM was pulled out of the midgut, then washed in sterile, pre-cooled, phosphate buffered saline solution (PBS, 140 mM NaCl, 1 mM KCl, 6 mM phosphate buffer, pH 7.4) to remove the food debris, and then flash-frozen in liquid nitrogen. Several PMs were immersed in paraformaldehyde for scanning electron microscopy observations.

### Total protein extraction

The total protein of the BSF larvae was extracted with protein lysis buffer (8 M urea with appropriate protease inhibitor), and treated twice with a high-throughput tissue crusher Wonbio-96c (Shanghai Wanbo Biotechnology Co., Ltd) for 40 s. The mixture was incubated on ice for 30 min, vortexed 5–10 s every 5 min, and then centrifuged at 16000×g at 4 °C for 30 min. Protein supernatants were mixed with four- fold volumes of cold acetone for 12 h to precipitate the protein and improve the final concentration. After centrifugation at 12000×g for 20 min, the pellet was rinsed twice with 90% acetone and dried under vacuum. The acetone dry powder was resuspended in protein lysis buffer (8 M urea with appropriate protease inhibitor), incubated on ice for 30 min, vortexed 5–10 s every 5 min, and then centrifuged at 16000×g at 4 °C for 30 min. Total protein concentration was determined by the bicinchoninic acid (BCA) method with a BCA Protein Assay Kit (Beyotime Biotechnology). Protein quantification was performed according to the kit protocol.

### Protein digestion

From the extracted proteins, in each group 80 μg protein were collected, and tetraethylammonium bromide (TEAB) was added to a final concentration of 100 mM. The mixture was reduced with 10 mM Tris (2-carboxyethyl) phosphine (TCEP) at 37 °C for 60 min and alkylated with 40 mM iodoacetamide (IAM) at room temperature for 40 min in darkness. Six- fold volumes of cold acetone were added to precipitate the protein at − 20 °C for 4 h. After centrifugation for 20 min at 10000×g at 4 °C, the pellet was rinsed with 90% acetone. Trypsin was added at a 1:50 trypsin-to-protein mass ratio and incubated at 37 °C overnight, then desalted with HLB and dried under vacuum.

### LC-MS/MS analysis

The trypsin-digested peptides were analyzed by online nano-flow liquid chromatography tandem mass spectrometry performed on an EASY-nLC system (Thermo, USA) connected to a Q Exactive HF-X Quadrupole Orbitrap mass spectrometer (Thermo, USA) through a nanoelectrospray ion source. Briefly, the C_18_-reversed phase column (75 μm × 25 cm, Thermo, USA) was equilibrated with solvent A (2% ACN with 0.1% formic acid) and solvent B (80% ACN with 0.1% formic acid). The peptides were eluted using the following gradient: 0–1 min, linear gradient of solvent B from 0 to 5%; 1–41 min, linear gradient of solvent B from 5 to 23%; 41–51 min, linear gradient of solvent B from 23 to 29%; 51–59 min, 29%–100% B; 59–65 min, gradient of solvent B 100%; 65–90 min, linear gradient of solvent B from 100 to 0%. The tryptic peptides were separated at a flow rate of 300 nL/min. The Q Exactive HF-X instrument was operated in data-dependent acquisition mode (DDA) to automatically switch between full scan MS and MS/MS acquisition. The survey of full scan MS spectra (m/z 350–1300) was acquired in the Orbitrap with 60,000 resolution. The automatic gain control (AGC) target was 3e6 and the maximum fill time was 20 ms. The top 20 most intense precursor ions were selected for fragmentation by higher-energy collision dissociation (HCD) in collision cells. The MS/MS resolution was set at 15000 (at m/z 100), the automatic gain control (AGC) target at 1e5, the maximum fill time at 50 ms, and dynamic exclusion at 18 s.

### Protein identification and bioinformatics analysis

The RAW data files were analyzed using ProteomeDiscoverer (Thermo Scientific, Version 2.2) against an in-house *H. illucens L* transcriptome database. The main MS/MS search parameters were as follows: Mass tolerance of 10 ppm for MS and 0.05 Da for MS/MS tolerance, trypsin as the enzyme with 2 missed cleavage allowed, carbamidomethylation of cysteine as fixed modification, and methionine oxidation as dynamic modifications. High confidence peptides were used for protein identifications by setting a target false discovery rate (FDR) threshold of 1% at the peptide level. Only proteins that had at least two unique peptides were used for protein identification. The preliminarily identified proteins were further analyzed through multiple databases, including the presence of signal peptides (SignalP http://www.cbs.dtu.dk/services/SignalP/), subcellular location (TargetP: http:// www.cbs.dtu.dk/services/TargetP/), motif analysis (https://myhits.sib.swiss/cgi-bin/motif_scan), and annotation based on the MEROPS database, UniProt database, and NCBI BLAST analysis. Gene ontology (GO) analysis and Kyoto Encyclopedia of Genes and Genomes (KEGG) analysis were performed using OmicShare tools, which is a free online platform for data analysis (www.omicshare.com/tools).

### Data availability

All raw LC-MS/MS data are available on the Mass Spectrometry Interactive Virtual Environment (MassIVE) data repository at ftp://massive.ucsd.edu/MSV000086950/

### Field emission scanning electron microscopy (FESEM)

The PM were immersed in paraformaldehyde for three hours. Then the PM were washed with PBS and dehydrated with a gradient alcohol series (30, 50, 70, 80, 90, 100%), 10 min each, repeated twice. After that, the PM were critical point dried with a FEI CPD-030 dryer and sputter-coated with 3–4 nm platinum. The morphological characteristics of the PM were observed under a Hitachi SU8010 scanning electron microscope (Hitachi, Tokyo, Japan).

## Results

### Identification of PM proteins

Due to the lack of complete *H. illucens* proteome data in the existing databases, we constructed an in-house EST contigs through transcriptome sequencing, and the raw data files from the LC-MS/MS were analyzed using ProteomeDiscoverer (Thermo Scientific, Version 2.2) against the derived protein sequences from the contigs.

Proteomic analysis identified a total of 565 proteins from the PM samples of *H. illucens* (Table S1). Since a few midgut epithelial cells may have been attached during the dissection of the PM, coupled with the high sensitivity of mass spectrometry, the results may have contained proteins from the epithelial cells. Therefore, we artificially screened the proteins to remove these. Since PM is a non-cellular structure, the proteins in the PM are mostly released from the microvilli of the columnar midgut cells into the extracellular space through apocrine secretion. Such secreted proteins usually contain a signal peptide sequence to guide them into the extracellular space, and usually do not contain a transmembrane domain. Some of these proteins did not have homologous proteins in public databases, and were thus suspected to be newly discovered proteins, for which further studies are needed to identify them. Combining the results of protein subcellular localization, a total of 88 proteins (Table 1) were identified as possible PM proteins.

**Table 1:** Proteins associated with the PM of *H. illucens* larvae

Contig name	Protein description^a^	*p*I	MW (kDa)	Main Feature
**1. Enzymes**				
**1.1 Protein metabolism**				
cds. TRINITY_DN12248_c0_g1_m.1812	Trypsin-2-like	5.07	28.1	Tryp_SPc
cds. TRINITY_DN13311_c0_g1_m.3204	Trypsin-1	4.92	27.2	Tryp_SPc
cds. TRINITY_DN14779_c0_g1_m.5149	Trypsin-7	5.43	28.6	Tryp_SPc
cds. TRINITY_DN13668_c0_g1_m.3716	Trypsin delta/gamma	6.04	28.6	Tryp_SPc
cds. TRINITY_DN18689_c0_g1_m.11218	Trypsin-like protease	9.31	24.9	Tryp_SPc
cds. TRINITY_DN15755_c2_g1_m.6527	Trypsin delta/gamma-like protein	5.85	26.0	Tryp_SPc
cds. TRINITY_DN11388_c0_g1_m.848	Serine protease	5.94	30.4	Tryp_SPc super family
cds. TRINITY_DN12733_c0_g1_m.2440	Serine proteases 1/2	5.6	31.2	Tryp_SPc
cds. TRINITY_DN17609_c0_g2_m.9414	Serine proteases 1/2	8.09	30.0	Tryp_SPc
cds. TRINITY_DN12169_c0_g1_m.1711	serine protease1/2	5.66	28.3	Tryp_SPc super family
cds. TRINITY_DN16378_c0_g2_m.7436	Serine protease	5.68	44.5	Tryp_SPc; CLIP_1
cds. TRINITY_DN15101_c0_g1_m.5599	Serine protease easter	6.42	41.8	Tryp_SPc; CLIP_1
cds. TRINITY_DN803_c0_g1_m.17778	Brachyurin-like	6.00	30.8	Tryp_SPc
cds. TRINITY_DN10692_c0_g1_m.300	Brachyurin-like	5.30	31.0	Tryp_SPc
cds. TRINITY_DN11863_c0_g1_m.1372	Chymotrypsin	5.87	27.4	Tryp_SPc
cds. TRINITY_DN17320_c1_g1_m.8906	Chymotrypsin BI	6.23	31.5	Tryp_SPc
cds. TRINITY_DN10739_c0_g1_m.333	Chymotrypsin-2	7.20	27.3	Tryp_SPc
cds. TRINITY_DN13390_c0_g1_m.3313	Chymotrypsin BI	5.77	32.2	Tryp_SPc
cds. TRINITY_DN10743_c0_g1_m.337	Chymotrypsin BI	5.31	31.7	Tryp_SPc
cds. TRINITY_DN21565_c0_g1_m.17048	Chymotrypsin BI	5.06	32.4	Tryp_SPc
cds. TRINITY_DN19986_c0_g1_m.13768	Carboxypeptidase B	6.57	45.9	Propep_M14
cds. TRINITY_DN19294_c0_g1_m.12373	Angiotensin-converting enzyme	5.20	73.6	Peptidase_M2
**1.2 Lipid metabolism**				
cds. TRINITY_DN13210_c0_g1_m.3048	Pancreatic triacylglycerol lipase	5.27	37.4	Pancreat_lipase_like
cds. TRINITY_DN13611_c0_g2_m.3637	Lipase	8.38	35.0	Pancreat_lipase_like
cds. TRINITY_DN16324_c0_g1_m.7354	Lipase	5.53	37.2	Pancreat_lipase_like
cds. TRINITY_DN13611_c0_g1_m.3635	Lipase	6.15	35.3	Pancreat_lipase_like
**1.3 Glycosyl hydrolase**				
cds. TRINITY_DN16881_c0_g3_m.8198	Alpha-amylase	6.47	53.8	Aamy_C
cds. TRINITY_DN18580_c0_g1_m.10995	Maltases	4.97	69.8	AmyAc_maltase
**1.4 Others**				
cds. TRINITY_DN11418_c0_g1_m.880	Chitinase-like protein	7.47	48.9	GH18_IDGF
cds. TRINITY_DN20328_c0_g1_m.14492	Sphingomyelin phosphodiesterase	5.83	70.3	MPP_ASMase; SapB
cds. TRINITY_DN14434_c0_g1_m.4713	Adenosine deaminase	5.62	62.8	Adm_rel super family
**2. Transporters**				
cds. TRINITY_DN15890_c0_g1_m.6730	Transferrin	6.18	78.8	PBP2_transferrin
cds. TRINITY_DN16029_c2_g1_m.6931	Ferritin	6.38	25.5	Euk_Ferritin
**3. Odorant-binding protein**				
cds. TRINITY_DN13988_c0_g1_m.4120	Odorant-binding protein	7.33	14.3	PBP_GOBP
cds. TRINITY_DN11658_c0_g1_m.1123	Odorant-binding protein	7.20	15.0	PBP_GOBP
cds. TRINITY_DN11155_c0_g1_m.640	Odorant-binding protein	7.65	15.8	PBP_GOBP
cds. TRINITY_DN11257_c0_g1_m.728	Odorant-binding protein	5.24	15.3	PBP_GOBP
cds. TRINITY_DN13076_c0_g1_m.2863	Odorant-binding protein	6.76	15.6	PBP_GOBP
cds. TRINITY_DN9614_c0_g1_m.18143	Odorant-binding protein	7.08	16.5	PBP_GOBP
cds. TRINITY_DN16710_c0_g8_m.7928	Odorant-binding protein	8.13	16.5	PBP_GOBP
cds. TRINITY_DN16710_c0_g3_m.7924	Odorant-binding protein	6.95	14.2	PBP_GOBP
**4. Immunity/defense**				
cds. TRINITY_DN16169_c3_g1_m.7152	SVWC domain-containing protein	5.08	15.8	SVWC
cds. TRINITY_DN16192_c2_g1_m.7187	Lysozyme c	8.31	14.8	LYZ_C_invert
cds. TRINITY_DN13537_c1_g3_m.3530	MD-2-related lipid-recognition protein	6.57	17.1	ML super family
cds. TRINITY_DN11748_c0_g1_m.1238	MD-2-related lipid-recognition protein	7.83	17.0	ML super family
cds. TRINITY_DN19333_c0_g1_m.12455	Hemocytin	5.69	435.7	5 × VWD; 5 × TIL; 2 × FA58C
cds. TRINITY_DN17913_c0_g1_m.9886	3-glucan binding protein	7.4	53.7	CBM39; GH16_beta_GRP
cds. TRINITY_DN18830_c0_g1_m.11490	Beta-1,3-glucan-binding protein	5.39	42.5	GH16_CCF
**5. Chitin binding**				
cds. TRINITY_DN19733_c1_g5_m.13257	Peritrophin-48-like	4.82	20.7	ChtBD2
cds. TRINITY_DN16786_c0_g1_m.8036	Peritrophin-48-like	4.68	34.8	ChtBD2
cds. TRINITY_DN18694_c0_g3_m.11232	Peritrophin-44	5.11	31.8	ChtBD2
cds. TRINITY_DN11775_c1_g1_m.1271	Peritrophin-48-like	5.53	35.1	2 × CBM_14
cds. TRINITY_DN17674_c0_g2_m.9518	Unnamed protein product	4.55	39.6	ChtBD2
cds. TRINITY_DN14642_c0_g4_m.4965	Peritrophic matrix protein 14	4.89	36.2	2 × CBM_14
cds. TRINITY_DN18631_c0_g1_m.11102	Uncharacterized protein	4.58	33.7	2 × ChtBD2; 3 × CBM_14
cds. TRINITY_DN19733_c1_g3_m.13254	Uncharacterized protein	5.3	36.8	2 × ChtBD2
cds. TRINITY_DN17744_c0_g2_m.9623	Peritrophin-44-like	4.93	36.0	ChtBD2
cds. TRINITY_DN16319_c1_g1_m.7350	Peritrophin-44	4.89	36.9	2 × ChtBD2
cds. TRINITY_DN12826_c0_g1_m.2569	Uncharacterized protein	6.81	16.7	CBM_14
cds. TRINITY_DN12769_c0_g1_m.2499	Uncharacterized protein	4.67	24.8	ChtBD2
cds. TRINITY_DN11775_c1_g2_m.1272	Unnamed protein product	4.64	33.5	2 × ChtBD2
cds. TRINITY_DN21192_c8_g1_m.16673	Uncharacterized protein	4.30	39.2	CBM_14
cds. TRINITY_DN12094_c0_g1_m.1629	Uncharacterized protein	7.69	15.0	CBM_14; ChtBD2
cds. TRINITY_DN13289_c0_g1_m.3171	Uncharacterized protein	7.71	15.0	CBM_14; ChtBD2
**6. Other protein**				
cds. TRINITY_DN20571_c0_g2_m.15053	Hexamerin	8.31	67.1	Hemocyanin_M; Hemocyanin_N; Hemocyanin_C
cds. TRINITY_DN19822_c1_g1_m.13436	Hexamerin	6.95	124.6	Hemocyanin_M; Hemocyanin_N; Hemocyanin_C
cds. TRINITY_DN19826_c0_g1_m.13447	Hexamerin	9.01	93.2	Hemocyanin_M; Hemocyanin_N; Hemocyanin_C
cds. TRINITY_DN17686_c0_g2_m.9540	Hexamerin 2 beta	6.55	84.1	Hemocyanin_M; Hemocyanin_N; Hemocyanin_C
cds. TRINITY_DN16262_c0_g1_m.7277	Hexamerin	6.37	83.1	Hemocyanin_M; Hemocyanin_N; Hemocyanin_C
cds. TRINITY_DN17535_c2_g2_m.9284	Laminin	5.43	413.1	LamG
cds. TRINITY_DN14691_c0_g1_m.5031	Calumenin	4.67	38.5	EFh_CREC_Calumenin_like
cds. TRINITY_DN13489_c0_g1_m.3457	Nidogen	4.78	147.6	EGF_3; LY
cds. TRINITY_DN18659_c0_g3_m.11159	Uncharacterized protein	4.53	107.7	5 × DUF753
cds. TRINITY_DN12676_c0_g1_m.2371	Uncharacterized protein	4.12	49.5	Tryp_SPc super family
cds. TRINITY_DN18428_c0_g1_m.10766	Uncharacterized protein	4.53	74.1	4 × DUF753
cds. TRINITY_DN4827_c0_g1_m.17337	Unnamed protein product	4.7	28.6	JHBP
cds. TRINITY_DN16431_c0_g1_m.7515	Uncharacterized protein	4.27	50.9	2 × DUF753
cds. TRINITY_DN11073_c0_g1_m.579	Uncharacterized protein	8.12	27.5	JHBP
cds. TRINITY_DN12629_c0_g1_m.2312	Uncharacterized protein	4.92	33.2	DUF1397
cds. TRINITY_DN17619_c0_g2_m.9425	Unnamed protein product	4.88	23.5	None
cds. TRINITY_DN13426_c0_g1_m.3363	Uncharacterized protein	4.77	24.4	None
cds. TRINITY_DN14613_c0_g1_m.4924	Uncharacterized protein	7.47	58.3	None
cds. TRINITY_DN18647_c0_g1_m.11141	Uncharacterized protein	4.55	74.3	3 × DUF753
cds. TRINITY_DN12553_c0_g4_m.2207	Unnamed protein product	4.65	17.4	None
cds. TRINITY_DN14642_c0_g5_m.4966	Unnamed protein product	4.86	18.8	None
cds. TRINITY_DN12917_c0_g1_m.2687	Uncharacterized protein	5.49	16.9	None
cds. TRINITY_DN16299_c0_g1_m.7327	Unnamed protein product	4.78	59.7	3 × EB
cds. TRINITY_DN15832_c0_g1_m.6643	Uncharacterized protein	5.29	53.7	NUC

*Tryp_SPc* Trypsin-like serine protease, *CLIP_1* Serine protease Clip domain PPAF-2, *Propep_M14* Carboxypeptidase activation peptide, *ChtBD2* Chitin-binding domain type 2, *CBM_14* Chitin binding Peritrophin-A domain, *MPP_ASMase* acid sphingomyelinase and related proteins, metallophosphatase domain, *SapB* Saposin (B) Domains, *Adm_rel super family* adenosine deaminase-related growth factor, *LYZ_C_invert* C-type invertebrate lysozyme, *DUF753* Protein of unknown function

^a^Annotation based on MEROPS database, UniProt database and NCBI BLAST analysis

### Functional annotation of PM proteins by GO and KEGG analyses

**Fig. 1:**
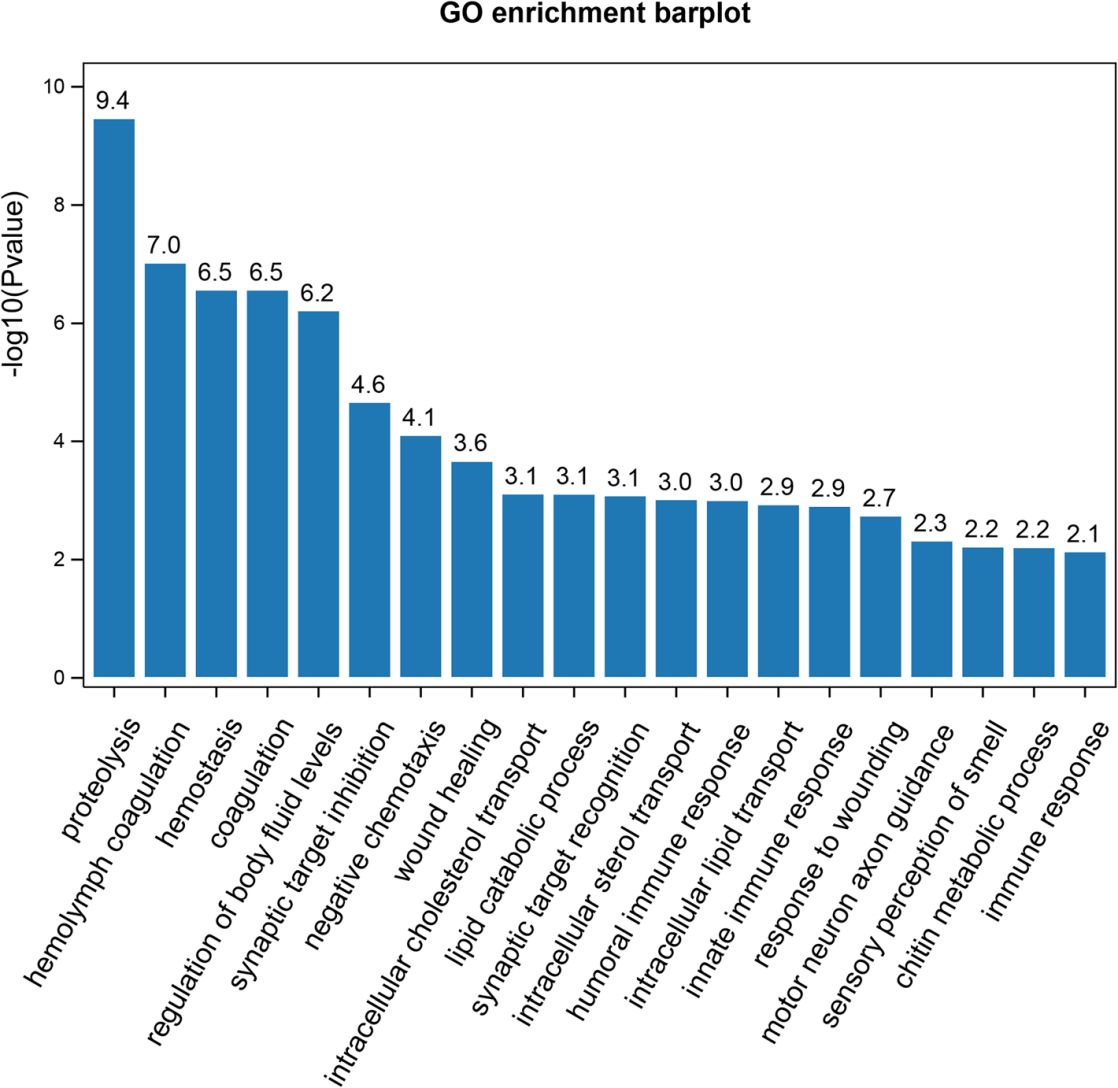
GO enrichment analysis of the predicted PM proteins. GO, Gene Ontology

**Fig. 2:**
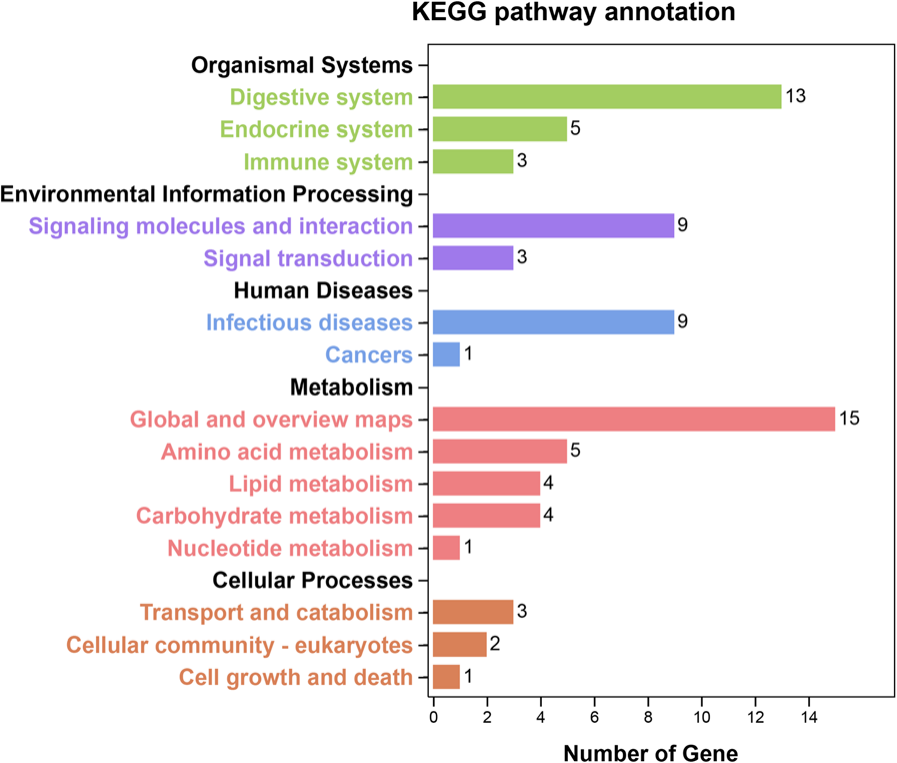
KEGG pathway enrichment of the predicted PM proteins. KEGG, Kyoto Encyclopedia of Genes and Genomes

Enrichment analyses were performed to elucidate the biological function of the PM proteins. The results of the GO analysis demonstrated that the enriched GO terms were ‘proteolysis (GO:0006508)’, ‘hemolymph coagulation (GO:0042381)’, ‘regulation of body fluid levels (GO:0050878)’, ‘wound healing (GO:0042060)’, ‘innate immune response (GO:0045087)’, ‘chitin metabolic process (GO:0006030)’ etc. (Fig. 1), demonstrating that most queried proteins were involved in biological processes related to metabolic processes, immunity and so on. KEGG analysis revealed that PM proteins were enriched in metabolism, organismal systems, Environmental information processing, human disease and cellular processes (Fig. 2).

### Ultrastructure of the PM

**Fig. 3:**
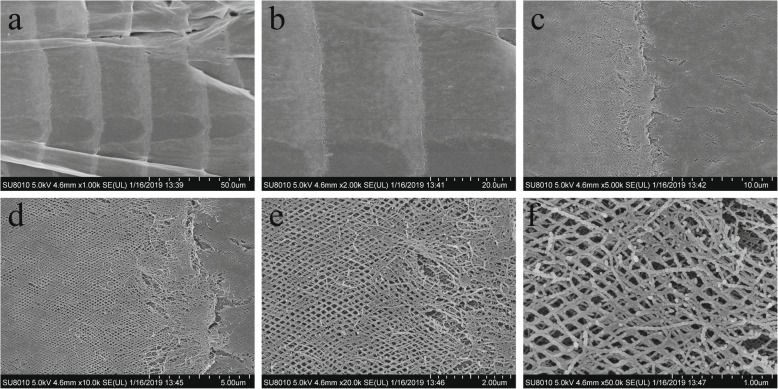
Morphology of *H. illucens* larval PM observed by SEM under different magnification. **a** was observed under ×1000 magnification, the outermost layer of the PM was arranged in continuous segments, and the length of each segment was approximately the same, about 25 μm; **b** and **c** was observed under ×2000 and ×5000 magnification respectively, the chitinous fibers at the anterior end of the outer PM layer are arranged loosely, and appears brighter, while chitinous fibers at the posterior end are densely arranged and is darker; **d** and **e** was observed under × 10, 000 and × 20, 000 magnification respectively, chitinous fibers cross each other into diamond-shaped grids, and are filled with various proteins; **f** The chitinous-protein fibers can be clearly observed under × 50, 000 magnification

The SEM results showed that the outermost layer of the PM was arranged in continuous segments, and the length of each segment was approximately the same, about 25 μm (Fig. 3a). At the junction of every two sections, chitinous fibers intersect each other in a grid shape. The fibers at the anterior end of the outer PM layer are arranged loosely and appear brighter, while fibers at the posterior end are densely arranged and darker (Fig. 3b, c). Under high magnification, the fibers cross each other into diamond-shaped grids, which are filled with various proteins (Fig. 3d, e). Under higher magnification, one sees that many proteins are embed in the chitin scaffold (Fig. 3f).

## Discussion

### Proteins involved in digestion and metabolism

One of the functions of the PM is to compartmentalize the midgut and divide it into the endoperitrophic, ectoperitrophic, and intraperitrophic spaces. After ingested food enters the midgut, digestive pro-enzymes are secreted from the anterior region of the midgut into the midgut lumen through extracellular secretion or apocrine secretion, then proteolytically activated. Digestion begins in the anterior region of the midgut as digestive enzymes cleave food macromolecules. Through peristaltic contraction of the PM, food and digestive enzymes (nucleases, lipases, proteases, etc.) are moved into the median midgut for further digestion. In the ectoperitrophic space, aminopeptidase, carboxypeptidase and amylase perform the final digestion. By compartmentalizing the midgut, damage to the enzyme catalytic sites and midgut epithelial cells caused by the accumulation of digestive intermediates are avoided, promoting food digestion [30–35].

In the putative PM proteome, we found twenty proteins belonging to the Chymotrypsin(S1) family, including six trypsin-like proteases, six serine proteases, two Brachyurin-like proteins, and six chymotrypsins. Trypsin is a kind of endoprotease that mainly cleaves the peptide chain at the carboxyl part of lysine or arginine. Trypsin is a major protease in the midgut of most insects (including Orthoptera, Hymenoptera, Diptera, Lepidoptera and Coleoptera) with a molecular weight of 20–35 kDa, an isoelectric point of 4–5, and an optimum pH range of 8–11 [36–38]. Brachyurin, also known as collagenase, was first found in the hepatopancreas of the fiddler crabs *Uca pugilator*. The site for cleavage of collagen is usually located 3/4 away from the amino end of the collagen chain. Brachyurin has a wide range of specificity for peptide bonds, but exhibits low activity for the main substrates of trypsin and chymotrypsin [39]. Chymotrypsin is a major digestive enzyme in the midgut of Diptera. Its cleavage site is usually located at the C-terminal peptide bond of a hydrophobic amino acid, and is more inclined to long-chain substrates. Chymotrypsin can catalyze the decomposition of proteins to produce free amino acids necessary for the growth and development of insects [40, 41]. There are two types of catalytically active chymotrypsin found in the midgut of the fruit fly. One is located in the midgut lumen with high catalytic activity, and the other is located in the cell membrane with weak catalytic activity [42]. Serine protease is the main digestive enzyme in insect midguts, and thus the target of most pest control strategies based on protease inhibitors [43]. In the course of evolution, plants produced some protease inhibitors to prevent insects from feeding, and insects correspondingly secreting excessive proteases or proteases that are not sensitive to these inhibitors. Therefore, in the past decades, a large number of protease-encoding genes were found in the digestive system of insects.

In the putative PM proteome, we found two metallopeptidases, one in the carboxypeptidase (M14) family, and one in the peptidyl-dipeptidase A (M2) family. Digestive carboxypeptidase is an exonuclease, which requires divalent metal cations (such as Zn^2+^) to catalyze the hydrolysis of the peptide bond at the carboxyl terminal of the polypeptide. The members of the carboxypeptidase M14 family can be divided into carboxypeptidase A and carboxypeptidase B according to the types of amino acids released by their catalytic functions. Carboxypeptidase A is more prone to hydrolyze polypeptides with aromatic or hydrophobic amino acids at the carboxyl terminal. Carboxypeptidase B is more inclined to hydrolyze peptides with lysine and arginine at the carboxyl terminal [44]. Besides digestion, carboxypeptidase can be involved in other physiological activities. For example, carboxypeptidase was found in the molting fluid of *Helicoverpa armigera* larvae and pre-pupa, and is believed to be related to apolysis [45]. Carboxypeptidase B in the midgut of *Anopheles gambiae* is related to the sexual development of *Plasmodium*, and is a potential target for malaria prevention and control [46]. One protein belonging to the peptidyl-dipeptidase A (M2) family was identified as angiotensin-converting enzymes (ACEs). In insects, ACE is involved in the metabolic inactivation of peptide neurotransmitters and processing prohormones into active peptide hormones [47–49]. ACE is expressed in many tissues in *Drosophila*, such as reproductive tissues, larval and adult midguts, larval tracheae, and adult salivary glands. ACE expression has also been detected in both sexes’ reproductive tissues in other insects, indicating that it plays an important role in reproduction [50–52].

Lipase plays an important role in the acquisition, storage, and metabolism of fat [53, 54]. In this study, a total of four lipases were found, including one pancreatic triacylglycerol lipase (TAG-lipase). TAG-lipases can hydrolyze the outer ester link of TAG and act on the water-lipid interface. Insects’ TAG-lipases need to be activated by calcium ions, and will preferentially hydrolyze unsaturated fatty acids, similar to mammalian pancreas TAG-lipases [55, 56]. Compared to mammals, few studies on insect pancreatic TAG-lipases exist, such as a pancreatic lipase-related protein as found in the brown planthopper, *Nilaparvata lugens*, that is essential for the hatching of eggs [57, 58]. A functional study using RNAi found that pancreatic TAG-lipases may be related to the virulence of BPH to rice varieties [59].

Carbohydrates are important energy sources in different developmental stages of insects. Three carbohydrases were found in this study: one α-amylases, and one maltase. In insects, the digestion of starch usually depends on these two enzymes. Maltose and glucose generated by amylase through hydrolyzing the α-1,4 glycosidic bond of starch are the energy sources for insect development, reproduction, and flight, with further digestion performed by maltase through hydrolyzing the α-1,4 or α-1,6 glycosidic bonds at the non-reducing ends of these oligosaccharides [60, 61]. Insect amylase relies on calcium and chloride ions to maintain activity and structural integrity, but it has also been reported that amylase in lepidopteran insects does not require chloride ions to activate [61, 62]. Maltase, also known as α-glucosidase, is a typical exo-type hydrolase, divided into family I and family II according to its structure. Insect α-glucosidase is classified as family I. Family I α-glucosidase has four conserved regions, which can also be found in α-amylase, but there is no sequence similarity between them [63]. Carbohydrases are often used as targets for pesticides, such as amylases or glucosidase inhibitors [64, 65].

We found a sphingomyelin phosphodiesterase (SMPD), an adenosine deaminase, and a chitinase-like protein in the PM proteome of *H. illucens*. SMPD is a hydrolase involved in the metabolism of sphingolipids. It can hydrolyze sphingomyelin into phosphocholine and ceramide, and plays an important role in signal transduction. In the bumble bee (*Bombus lantschouensis*), SMPD is expressed in various tissues, with higher expression levels in the ovary and midgut [66]. Differential expression of its homologous protein in *Anopheles gambiae* infected by different bacteria suggests it may also be involved in invertebrate immune response [67, 68]. Chitinase belongs to family 18 of the glycoside hydrolases, and can hydrolyze chitin into small, soluble oligosaccharides. Chitinase is very important for the regulation of the thickness and permeability of the PM, and is also involved in the degradation of the PM during molting. The products of chitin hydrolysis can be recycled to synthesize new chitin [69].

### Transporter

A transferrin and a ferritin were found in the PM proteome of *H. illucens*. Iron participates in a variety of physiological activities, such as oxygen metabolism, amino acid production, and DNA biosynthesis. In some arthropods, it also participates in egg development and offspring production [70, 71]. However, iron can also cause production of reactive oxygen species and highly reactive radicals, which may cause cell death and tissue damage. Therefore, vertebrates and arthropods have evolved a variety of specific proteins, such as transferrin and ferritin, to keep iron in a safe form [72]. Transferrin is a secreted protein with high affinity for iron ions. In insects, it not only participates in iron transport, but also has some immune functions [71, 73, 74]. Ferritin is a sphere complex with 24 subunits, which can hold thousands of iron atoms and maintain a nontoxic state. It is the main protein that stores iron in organisms [75]. For insects, the secretion of iron-laden ferritin through the midgut and Malpighian tubules is the main way for insects to excrete iron [76].

### Proteins involved in signaling

We found eight odorant-binding proteins (OBP) in the PM proteome of *H. illucens*. OBPs are soluble proteins with a small molecular weight, usually between 17 and 22 kDa, first found in the antennae of the male giant moth (*Antheraea polyphemus*). They can bind, solubilize, and deliver odor molecules or pheromones to their receptors [77–79]. There exists a relatively conserved sequence in insect OBPs, which contains six cysteine residues and can form three disulfide bonds, which also makes the tertiary structure of OBP conserved. The number of OBP genes in different insect genomes varies from a few to hundreds [80–82]. In addition to their abundant expression in insect antennae, some OBP family members are expressed in other tissues, such as the abdomen and wings of *Periplaneta americana* [83], and the midgut and head of diamondback moths (*Plutella xylostella*) [84]. Four OBPs in honeybees are similarly highly expressed in regions with low chemosensory receptor expression [85]. These cases indicate that OBP may participate in other life activities besides chemosensation, such as responses to adverse environmental conditions and the scavenging of various, small, hydrophobic molecules [86, 87].

### Proteins involved in larval innate immunity

BSF is an omnivorous insect with a wide range of larval diets, so its food may carry various bacteria, viruses, fungi, or parasites that can infect the insect when it eats. In the past few decades, there have been many studies on how insects fight these infections, and the PM is considered to be the first line of defense [3]. In fact, early reports hypothesized that PM production was primarily to protect midgut epithelial cells from infection by pathogens in food [69]. In addition to the aforementioned immune-related serine proteases and other proteins, some other proteins related to larval immunity were also found in this study, including two beta-1,3-glucan-binding protein (BGRP), one protein with an SVWC domain, one C-type lysozyme, two MD-2 related lipid recognition proteins and one hemocytin. The insect innate immune system uses a large number of pattern recognition proteins to recognize the molecular characteristics of pathogenic microorganisms, thereby triggering a series of immune defense mechanisms. BGNP is a main pattern recognition receptors in insects and is more specific to fungi [88]. SVWC is a short-sequence protein in arthropods that can respond to changes in nutritional status or environmental changes such as bacterial and viral infections. In the European bumblebee (*Bombus terrestris*), SVWC participates in the host’s antiviral immunity and is related to the expression of antimicrobial peptides [89]. Lysozyme is an antibacterial enzyme widely found in bacteriophages, plants, and animals. It can dissolve bacteria by hydrolyzing the cell walls of bacteria. Animal lysozymes are divided into c-type, g-type and i-type, with c-type lysozyme expression affected by bacterial challenge [90]. MD-2 related lipid recognition protein is a type of secretory or luminal protein that binds to lipids and is mainly expressed in the gut. It can also bind to bacterial lipopolysaccharides and interact with cell receptors, which are important for innate immunity [91]. Hemocytin is a protein in insects homologous to mammalian von Willebrand factor. The expression of hemocytin in the silkworm is up-regulated following bacterial infection and before pupation, indicating it may play an important role in immunity and metamorphosis [92].

### PM proteins with chitin-binding domains

In addition to the above-mentioned, non-structural proteins, some structural proteins, namely peritrophin, were found in the PM proteome of *H. illucens*. Peritrophins usually contain more than one chitin-binding domain (CBD), which are called the peritrophin-A domain (PAD), peritrophin-B domain, and peritrophin-C domain with 6, 8, or 10 cysteine residues respectively that can form 3–5 disulfide bonds, of which PAD is the most common (2). PAD is also called type-2 chitin-binding domain (ChtBD2), and its sequence feature is CX_13–20_CX_5–6_CX_9–19_CX_10–14_CX_4–14C_, where X represents any amino acid except cysteine [2, 93]. PAD is mainly found in peritrophin, but it is also found in midgut chitinase, as well as some proteins in the Malpighian tube, rectum, and epidermis [94, 95]. Peritrophin-44 is the first peritrophin found in the PM of *Lucilia cuprina* larvae with several CBDs. These tandem CBDs may facilitate cross-linking chitin fibrils within the PM. Peritrophin-44 and another protein, Peritrophin-48, are the main proteins involved in the construction of PM, and represent approximately 70% of the protein mass of the PM [96–99]. In this study, in addition to Peritrophin-44 and Peritrophin-48, we also found a variety of unknown proteins containing ChtBD2 domains. The functions of these proteins need further study.

### Other PM proteins

Among the other proteins in the BSF PM proteome are hexamerins. Hexamerins can store a large amount of amino acids, which play an important role in insect metamorphosis and reproduction, and when suffering adverse environments such as food shortages [100]. Hexamerin is expressed in all insect life stages, reaching a peak in the last instar. Hexamerin is usually expressed in the fat body and stored in the hemolymph, but is also moderately expressed in the midgut of *Apriona germari*, and in the midgut, epidermis, and Malpighian tubules of *Spodoptera exigua* [101, 102]. Expression of hexamerin in tussah silkworm (*Antheraea pernyi*) was significantly increased when they were infected with microorganisms, so it may be related to immune response [103].

A large number of uncharacterized or unnamed proteins were also discovered. These proteins should not be ignored, and more research is needed on them, because they may have important significance to the molecular architecture of the PM or lead to better breeding and utilization of BSF larvae.

We also compared the identified PM proteome of *H.illucens* with other reported insects, especially the *Musca domestica* which also belongs to Diptera. In terms of the number of proteins identified, there are 47 species in the 5th instar *Bombyx mori* [18], 71 secreted proteins in *M.domestica* [104], and 115 in *Aedes aegypti* [105]. From the point of view of protein function, the PM proteome of different insects are all involved in the physiological processes of digestion, metabolism and immunity. In terms of digestion, trypsin, amylase, and carboxypeptidase were found in the PM proteome of *H.illucens* and *M.domestica* [104]. In addition, some Brachyurin-like proteins which can participate in the metabolism of collagen were also found in *H.illucens*. Peritrophin is a kind of important protein in the PM proteome. Only 4 peritrophin are found in *A.aegypti* [105], 8 in *M.domestica*, and 16 in *H.illucens*. This may be due to the PM of *A.aegypti* is usually induced following a blood meal. While the larva of *M.domestica* and *H.illucens* constantly feed before pupation, but the instar of *H.illucens* is much longer than that of *M.domestica*. From the molecular weight point of view, most of the peritrophin are between 20 and 40 kDa, and a number of peritrophin of about 16 kDa have been found in *H.illucens*. The function of these peritrophin needs further verification.

### Ultrastructure of the PM

The SEM results showed that the outermost layer of the PM was arranged in continuous segments, and the length of each segment was approximately the same, about 25 μm (Fig. 1a). It was observed in well-fed *Locusta migratoria,* the midgut secretes PM every 15 min. The inner PM will move posteriorly with the food, making the PM appear as a telescope-like structure (3), as we observed in the BSF PM (Fig. 1b, c). A grid-like structure of chitin fibers observed in the BSF PM (Fig. 1d, e) may be found in the PM of other insects, but the currently known arrangements are very different. For example, the PM from larval *Trichoplusia ni* is organized in a random, felt-like structure of fibers [106], the *Ostrinia nubilalis* PM in an orthogonal structure of fibers [107], and *Anomala cuprea* PM in a hexagonal structure of fibers. The significance of the differences in arrangement are not clear yet. We speculate that it may be related to the feeding habits of the insects. BSF larvae are described as voracious feeders in most studies, and a diamond-shaped chitin grid is more flexible when deformed, so it may enable their PM to hold more food.

## Conclusions

The main function of the PM is to protect the midgut epithelial cells from coarse food particles, pathogens, and toxins, and it can also compartmentalize the midgut to promote digestion. In this study, we discovered a large number of digestive enzymes bound to the BSF PM, which can digest a variety of proteins, fats, and other ingested food particles. We also discovered a variety of immune-related proteins known to play roles in the identification of and defense against pathogens. The findings of some transporters suggest that the PM maybe involved in ion transport and excretion. The findings of some odorant-binding proteins also indicate that the PM is an important place for insects to interact with their external environment. Scanning electron microscopy revealed clearly the ultrastructure of the BSF PM, and found that it is a scaffold that can hold a variety of proteins. PMs in insects display diverse textures on this ultramicro scale, but the reason for this diversification needs further study. To summarize, our work used liquid chromatography-tandem mass spectrometry and scanning electron microscopy to conduct a more in-depth study of the PM of BSF larvae, and deepen our understanding of its molecular architecture and ultrastructure.

## Supplementary Information

**Additional file 1: Table S1**. all the identified protein from peritrophic matrix sample

## Data Availability

All data generated or analysed during this study are included in this published article and its supplementary information files or from the corresponding author on reasonable request.

## Abbreviations

BSF: Black soldier fly
PM: Peritrophic matrix
SEM: Scanning electron microscopy
CBD: Chitin-binding domain
BCA: Bicinchoninic acid
TCEP: Tris (2-carboxyethyl) phosphine
IAM: Iodoacetamide
DDA: Data-dependent acquisition mode
AGC: Automatic gain control
HCD: Higher-energy collision dissociation
FDR: False discovery rate
FESEM: Field emission scanning electron microscopy
S1 family: Chymotrypsin family
M14 family: Carboxypeptidase family
M2 family: Peptidyl-dipeptidase A family
ACE: Angiotensin-converting enzymes
TAG: Pancreatic triacylglycerol
SMPD: Sphingomyelin phosphodiesterase
OBP: Odorant-binding proteins
BGRP: Beta-1,3-glucan-binding protein
PAD: Peritrophin-A domain
ChtBD2: Type-2 chitin-binding domain
